# The Vestfold Hills are alive: characterising microbial and environmental dynamics in Old Wallow, eastern Antarctica

**DOI:** 10.3389/fmicb.2024.1443491

**Published:** 2024-09-23

**Authors:** Devan S. Chelliah, Angelique E. Ray, Eden Zhang, Aleks Terauds, Belinda C. Ferrari

**Affiliations:** ^1^School of Biotechnology and Biomolecular Sciences, The University of NSW, Kensington, NSW, Australia; ^2^Sydney Informatics Hub, Core Research Facility, University of Sydney, Sydney, NSW, Australia; ^3^Australian Antarctic Division, Department of Climate Change, Energy, the Environment and Water, Kingston, TAS, Australia; ^4^Evolution and Ecology Research Centre, The University of NSW, Kensington, NSW, Australia

**Keywords:** Antarctica, soil, eukaryotes, microbial biodiversity, Old Wallow, gradient forest

## Abstract

Old Wallow is an underexplored, hyper-arid coastal desert in Antarctica’s Vestfold Hills. Situated near an elephant seal wallow, we examined how stochastic nutrient inputs from the seal wallow affect soil communities amid environmental changes along a spatially explicit sampling transect. We hypothesized that nutrient levels would be elevated due to proximity to the seal wallow, influencing community distributions. While the soil bacterial and eukaryotic communities at the phylum level were similar to other terrestrial environments, analysis at class and family levels revealed a dominance of unclassified taxa that are often linked to marine environments. Elevated nutrient concentrations (NO_3_^−^, SO_4_^2−^, SO_3_) were found at Old Wallow, with conductivity and Cl^−^ levels up to 10-fold higher at the lowest elevation soils, correlating with significantly (*p* < 0.05) higher abundances of halophilic (*Halomonadaceace*) and uncultivated lineages (*Ca* Actinomarinales, unclassified *Bacillariophyta* and unclassified *Opisthonkonta*). An improved Gradient Forest model was used to quantify microbial responses to 26 soil gradients at OW, revealing variable responses to environmental predictors and identifying critical environmental thresholds or drivers of community turnover. Major tipping points were projected for eukaryotes with SO_4_^2−^, pH, and SO_3_, and for bacteria with moisture, Na_2_O, and Cl^−^. Thus, the Old Wallow ecosystem is primarily shaped by salt, sulphate, and moisture and is dominated by uncultivated taxa, which may be sensitive to environmental changes once critical tipping points are reached. This study provides critical baseline data for future regional monitoring under threats of environmental change.

## Introduction

Antarctica’s terrestrial biodiversity is concentrated within ice-free regions where continental soils are exposed. These ice-free regions, which account for about 0.35% of the total Antarctic landmass (Bockheim, 2015; Brooks et al., 2019), are characterised by extreme cold, desiccation, oligotrophy, high UV radiation, and frequent freeze–thaw cycles (Convey et al., 2018). Molecular methods and next-generation sequencing techniques continue to uncover an unexpectedly wide diversity of soil microorganisms on the continent, providing a critical source of baseline data (Cary et al., 2010; Lambrechts et al., 2019; Varliero et al., 2024). At a regional level, arid—hyperarid polar desert soil communities are like temperate soils, dominated by the bacterial phyla *Proteobacteria*, *Actinobacteria*, *Acidobacteria* and *Bacteroidetes* (Delgado-Baquerizo et al., 2018; Varliero et al., 2024). However, uncultured and taxonomically unique communities have been widely discovered including those containing high abundances of usually rare phyla, such as *Eremiobacterota* and *Ca. Dormibacterota* in the Windmill Islands (Ji et al., 2015; Ji et al., 2017) and *Patescibacteria* in the Larsemann Hills region of eastern Antarctica (Lambrechts et al., 2019).

Whilst Antarctic soil bacterial diversity and associated biogeography studies are widespread, knowledge of eukaryotic diversity is limited. In recent years, a high diversity of fungi, algae, and phagotrophic protists within Antarctic soil ecosystems have been described (Baeza et al., 2017; Thompson et al., 2020; Carvalho-Silva et al., 2021). Like their bacterial counterparts, high throughput sequencing has revealed a high diversity of uncultivated lineages (Gokul et al., 2013; Zhang et al., 2020), presenting the opportunity to discover novel species with distinctive metabolic capabilities. Furthermore, eukaryotes perform critical but overlooked roles within Antarctic soil communities. For example, in the McMurdo Dry Valleys, predatory protists, such as *Cercozoa*, are highly abundant and graze on bacterial populations (Thompson et al., 2020). Bacterial grazing is an integral part of the microbial food web, facilitating the release of cellularly trapped nutrients (Sherr, 2016). The same study also uncovered a high diversity of phototrophic protists, including *Chlorophyta* and *Bacillariophyta* (diatoms), known primary producers that use photosynthesis to assist in the production of soil organic carbon (Liao et al., 2023).

We now know that rare taxa and uncultivated lineages contribute significantly to total microbial diversity at lower taxonomic levels (Lambrechts et al., 2019; Almela et al., 2022). For example, *Chloracidobacteria,* a rare genus of *Acidobacteria* that is capable of photoheterotrophy using distinctive bacteriochlorophylls (Bchl a and c), dominates soil bacterial communities at proportions as high as 32% (Tytgat et al., 2016). Similarly, the genus *Crossiella*, within *Actinobacteria* has been detected in high relative abundances in eastern Antarctica, with their genomes found to contain biosynthetic gene clusters that produce potentially novel antimicrobials (Takahashi et al., 1986; Benaud et al., 2022). Despite these findings, eukaryotic diversity and function within Antarctic soil is disproportionately understood compared to bacterial taxa. Furthermore, as most studies investigate biodiversity at phylum level, there is a strong interest in developing baseline data on Antarctic terrestrial microbiomes at finer taxonomic scales. Such baseline data is timely, as preliminary studies have indicated that with projected warming—increasing thaw events and elevated moisture availability are expected to cause phylum level shifts in community taxonomies and biochemical cycles, posing a threat to the carbon balance and the uncharacterised communities within these sensitive environments (Zhang et al., 2024).

The Vestfold Hills is a coastal region in east Antarctica known as an Antarctic oasis (Bowman et al., 2000). With pockets of permanently ice-free patches, this region experiences less severe weather compared to other better-described east Antarctic regions (Seppelt and Broady, 1998; Cary et al., 2010; Zhang et al., 2020). The Vestfold Hills is a 400 km^2^ region formed by retreating ice during the last glacial–interglacial transition and is characterised by dry, rocky hills interspersed with >300 hypersaline lakes, including the largest number of meromictic lakes in the world (Seppelt and Broady, 1998). The average temperatures in the Vestfold Hills reach >2°C in the summer and fall as low as −40°C in the winter (Summerson and Bishop, 2011). Most biodiversity surveys in the region have focused on lithic and sub-lithic algae, mosses and lichens and microbial communities of hypersaline lakes (Bishop et al., 2020; Panwar et al., 2020). The few studies that have explored terrestrial biodiversity across the Vestfold Hills have revealed a dominance of *Actinobacteria*, *Proteobacteria* and *Bacteroidetes*, as well as the eukaryotic phyla—*Ochrophyta* and *Chlorophyta* (Line, 1988; Zhang et al., 2020), with the capacity for soil ecosystems to be supported by atmospheric chemosynthesis alongside photosynthesis (Ray et al., 2022). However, these investigations did not explore microbial community variation at a local scale, with few considerations of eukaryotic communities.

Old Wallow (OW), a coastal site in the Vestfold Hills is named after its proximity to a southern elephant seal (*Mirounga leonina*) wallow (Gales and Burton, 1989). The wallow measures ~1,000 m^2^, with clear evidence of moulting through the presence of seal fur, hair and faeces (Reynolds, 2009; van den Hoff, 2023). In the extreme cold Antarctic environment, seal and penguin matter decomposition occurs slowly, leading to higher nutrient inputs (Zvěřina et al., 2017), with seal carcasses on the continent previously dated from 250 to 2,600 years old (Pewe et al., 1959; Tiao et al., 2012). The OW sampling site is located 715 m east of a wallow, suggesting soils receive nutrient inputs not only from sea spray, given its location on the coast, but also from remnants of the wallow, which are known to impact microbial diversity and nutrient cycling (Tiao et al., 2012; Ball et al., 2015; Guo et al., 2018). Here, we aimed to characterise the microbial ecology of OW soils by investigating eukaryotic and bacterial community compositions along a spatially explicit sampling transect. Gradient Forest (GF) models are a combination of random forest models—allowing for quantification of the relative importance of various predictors with community compositional turnover along a suite of important environmental gradients (Stephenson et al., 2018; Zhang et al., 2024). We implemented an improved GF model suitable for large sequencing datasets to identify critical environmental thresholds alongside biotic and abiotic drivers influencing community turnover. We hypothesised that nutrient levels associated with the site’s proximity to an elephant seal wallow would be elevated and driving community distributions. Together, this baseline data will be helpful for future monitoring of the region, which is at risk from environmental change.

## Materials and methods

### Study area, soil sampling and physicochemical analysis

Sampling was performed by expeditioners via the Australian Antarctic Program at the Old Wallow site (68°36’S, 77°58′E) within the Vestfold Hills region (Supplementary Figure S1A). In total, 93 samples were aseptically collected from the 2–10 cm depth of the soil profile, along three parallel transects (T1, T2 and T3) spaced 3 m apart and measuring 300 m in length (Supplementary Figures S1B1, B3) (Siciliano et al., 2014; Zhang et al., 2020). Elevation varied across each transect, from 16 to 18 m, with the lowest point occurring in the middle of the sampling site (Supplementary Figure S2). The soil samples obtained were field-sieved to 2 mm. For each transect (i.e., T1, T2 and T3), 31 samples were retrieved, with each transect serving as biological replicates at each distance position. Distances between samples along a single transect ranged between 0.1 and 50 m (Supplementary Figure S1B2) (Siciliano et al., 2014). For downstream analysis, transects were clustered into three spatial groups comprising 31 samples according to distances as follows: start = 0–50 m, mid = 100–150 m, and end 200–300 m. The start of the transect (0 m) was closest to the coast and located approximately 715 m from a regularly populated elephant seal wallow (Supplementary Figure S1B1).

All soils included in this study were extensively physicochemically analysed according to previous studies (Siciliano et al., 2014; Zhang et al., 2020). Soil physicochemical parameters quantified include macronutrients (total carbon, total nitrogen and total phosphorous), water extractable ions (Cl^−^, NO_2_^−^, NO_3_^−^, PO_4_^3−^_,_ and SO_4_^2−^), metal oxides (SiO_2_, TiO_2_, Al_2_O_3_, Fe_2_O_3_, MnO, MgO, CaO, Na_2_O, K_2_O, SO_3_ and P_2_O_5_), dry matter fraction (DMF), conductivity, aspect, pH, and geographical parameters (latitude, longitude, slope, elevation, and aspect) (Supplementary Table S1) and the mean and standard error of all 31 soils within each distance group were reported (Table 1).

**Table 1:** Measured soil environmental parameters.

		0–100^a^			100–200^a^			200–300^a^		
		Mean	Std	Median	Mean	Std	Median	Mean	Std	Median
Geographical parameters	DMF	0.94	0.04	0.94	0.89	0.009	0.89	0.93	0.03	0.92
	Conductivity	4,246	4,091	3,355	14,270	2,274	14,553	1,326	1,085	1,088
	MSL^b^	17.61	0.6	17.9	15.62	0.1	15.59	17.22	0.64	16.9
	pH	8.66	0.57	8.5	8.3	0.26	8.4	8.11	0.75	7.9
Macronutrients (mg/kg)	Total C	6,727	1,165	2,919	3,585	671	3,663	2,387	1,651	1,647
	Total N	255.54	237.94	165	222.33	30.93	210	306.97	199.57	220
	Total P	749	263	700	636	38	630	672	62	680
Water extractable ions (ppm)	Cl^−^	4,337	5,528	2,795	20,747	3,687	20,550	1943	1,597	1,510
	NO_3_^2−^	14.81	13.81	8.93	3.34	4.94	1.8	4.23	3.9	3.1
	PO_4_^3−^	1.38	0.84	0.6	0	0	0	1.43	0.74	0.6
	SO_4_^2−^	2,916	3,640	983	725	207	735	137	84	117
Metal oxides (%)	SiO_2_	57.28	6.68	58.88	60.66	0.56	60.56	60.4	1.34	60.5
	TiO_2_	0.89	0.15	0.87	0.78	0.03	0.78	0.9	0.092	0.9
	Al_2_O_3_	13.02	1.65	13.58	12.62	0.28	12.56	13.83	0.23	13.82
	Fe_2_O_3_	10	0.93	9.85	8.23	0.28	8.18	10.14	1.05	10.18
	MnO	0.15	0.03	0.15	0.12	0.006	0.12	0.14	0.02	0.13
	MgO	5.4	1.31	4.96	4.81	0.1	4.84	4.92	0.39	4.95
	CaO	6	2.9	5.17	5.2	0.23	5.26	4.68	0.28	4.7
	Na_2_O	2.95	0.58	2.98	4.04	0.29	3.99	3.02	0.23	3.03
	K_2_O	1.55	0.39	1.46	1.62	0.08	1.61	1.69	0.18	1.68
	P_2_O_5_	0.2	0.07	0.19	0.18	0.006	0.18	0.18	0.01	0.18
	SO_3_	0.66	1.52	0.13	0.087	0.02	0.09	0.04	0.01	0.04

a The mean and SD are calculated for *n* = 31 soil samples for each transect section.

b Mean sea level.

### DNA extraction and illumina amplicon sequencing

Soil samples (0.25 g) were extracted and quantified in triplicate using the FASTDNA™ SPIN Kit for Soil (MP Biomedicals, Santa Ana, CA, US) and Qubit™ 4 Fluorometer (ThermoFisher Scientific, NSW, Australia) as per manufacturers’ instructions. Diluted DNA (1:10 using nuclease-free water) was submitted to the Ramaciotti Centre for Genomics (UNSW Sydney, Australia) for amplicon paired-end sequencing on the Illumina MiSeq platform (Illumina, California, United States). We targeted the 16S (27F/519R) and 18S rRNA gene sequences 1391f /EukBr using controls and preferred protocols used in the AusMicrobiome project (Bissett et al., 2016).

### Amplicon sequence data processing

Forward and reverse primer sequences were removed from the raw fastq files using cutadapt v2.10 (Martin, 2011). The trimmed paired-end reads were merged using FLASH 2 (Magoč and Salzberg, 2011) and then converted into FASTA format using SEQTK.^1^ Individual files were concatenated and imported into MOTHUR (Schloss et al., 2009) to screen the sequences and remove ambiguous bases and those with homopolymer runs >8 bp. The UPARSE/UNOISE2 algorithm (Edgar, 2013, 2016) was then implemented to dereplicate and denoise the screened sequences to generate a set of zero distance operational taxonomic units (zOTUs) and abundance count tables. For 16S rRNA amplicon gene datasets, taxonomy was classified against the SILVA v132 SSU rRNA database (Quast et al., 2013). For 18S rRNA amplicon gene datasets, taxonomy was classified against the Protist Ribosomal database 2 (PR2) (Guillou et al., 2013).

### Multivariate and statistical analyses in R

All multivariate and statistical analyses were conducted in the R environment (R Core Team, 2018) using 16S and 18S rRNA amplicon gene datasets for bacteria and eukarya. All plots were visualised using a combination of ggplot2 v3.1.0 (Wickham, 2011) and ggpubr v0.2 (Kassambara, 2023). Rarefaction curves (q = 0) were generated using the iNEXT package (Hsieh et al., 2016). The determination of differential abundant bacterial phyla between Old Wallow transect communities was performed using analysis of composition of microbiomes with bias correction (ANCOM-BC) (Lin and Peddada, 2020). ANCOM-BC estimates a change between test groups for each taxon using log-transformed values of absolute sequence counts. Samples were pooled by transect location (e.g., T1_100, T2_100 and T3_100), and all phyla over 0.5% relative abundance included in analysis. Results were corrected for multiple comparisons using the Holm-Bonferroni method, controlling the false discovery rate, and statistically valid results were included in the analysis (adjusted *p*-value <0.05) (Supplementary material S2). Alpha diversities were calculated using Vegan, including Simpson’s diversity, Shannon diversity, evenness, and richness (Dixon, 2003). ANOSIM statistics were generated using the package Vegan (version 2.6–4) using Euclidean distances and 9,999 permutations. Spearman correlations between key microbial representatives and physicochemical conditions were displayed using the R package “corrplot” (Wei and Simko, 2021) to determine the direction of correlations observed.

### Covariate network analysis on bacterial and eukaryotic communities

zOTUs representing over 0.00025% of the total relative abundance of the bacterial and eukaryotic communities were combined for network analyses alongside 29 physicochemical parameters to explore critical components of microbial networks. Correlations between the relative abundance of each zOTU pair across samples were calculated using the maximal information coefficient (MICe) in the MICtools software package using default parameters (Reshef et al., 2011). MICtools was also used to calculate Pearson, Spearman and TICe *p*-values to explore relationship characteristics further. Co-occurrence networks were visualised using Gephi software, displaying only very strong associations (MICe >0.8, *p* < 0.05) (Bastian et al., 2009). Network statistics were conducted using Gephi’s in-built statistical modelling. Node sizes were attributed to betweenness centrality, node colour associated with modularity (modularity is a measure of the structure of a graph, measuring the density of connections within a module or community) and connections coloured in association with positive and negative correlations (Pearson).

### Gradient forest modelling of microbial communities and physicochemical gradients

Gradient Forest (GF) analysis was conducted as per Zhang et al. (2024) on 1,387 eukaryotic and 10,864 bacterial zOTUs as well as all 29 physicochemical factors measured for 93 soil samples (Supplementary Table S1, Supplementary material S3). In brief, all multivariate and statistical analyses were carried out in the R environment (R Core Team, 2018). All plots were visualised using a combination of ggplot2 v3.1.0 (Wickham, 2011) and ggpubr v0.2 (Kassambara, 2023). For GF modelling, datasets were stored as individual phyloseq objects (Zhang et al., 2024). These were prepared by normalising the abundance data, agglomerating taxa at the phylum level, and extracting defined metadata variables as individual list elements for binary conversion and/or the removal of highly co-correlated variables (R > 0.7). The R package (Ellis et al., 2012) was used to fit the model (rp1 = 1,000, corr.threshold = 0.8) to our optimised list object and return a gradientForest object, which was used to generate a series of plots. These include (1) predictor overall importance plot, (2) splits density plot (shows where important changes or ‘splits’ in the abundance of multiple species are occurring along the gradient) and (3) predictor cumulative plot (shows the cumulative change in the overall composition of the community along the gradient). The predictor cumulative functions were also used to transform grid data layers of environmental variables to a common biological importance scale, which can be mapped onto biological and geographic space in a manner analogous to ordination whilst considering the non-linear and threshold changes that occur along gradients.

## Results

### Significant spatial variation of measured soil physicochemical parameters at the local scale

Variation in measured physicochemical properties was observed across the Old Wallow sampling transects (Table 1, Supplementary material S1). Soil moisture levels (DMF-1) varied between an average of 0.852–0.997%, with the highest moisture content detected in soils sampled from the middle of the transect. These variations occurred primarily on a small-distance scale of <100 m, with significant variation in moisture observed between the start and middle (R = 0.45, *p* = 0.0001) and between the middle and end (R = 0.46, *p* = 0.0001) of the sampling transects (Table 2). Notably, middle soils were also present at the lowest elevation point, approximately 2 m below the remaining sites along the OW site (Supplementary Figures S1, S2). Water soluble macronutrients were significantly (*p* < 0.05) higher at the start of the transects, closest to the elephant seal wallow (Supplementary Figure S1). For example, SO_4_^2−^ was 4.0-fold (R = 0.30, *p* = 0.0001) and 21.3-fold (R = 0.82, *p* = 0.0001) higher at the start than in the middle and end of the transects, respectively (Tables 1, 2). NO_3_^−^ ranged from 3–5 to 4.4-fold (R = 0.18, *p* = 0.0001; R = 0.05, *p* = 0.0163) higher, and SO_3_ was 7.6-fold (R = 0.25, *p* = 0.0001) and 16.5-fold (R = 0.77, *p* = 0.0001) higher at the start of the transects. In contrast, several parameters were significantly (*p* < 0.05) higher in the middle of the transect, these were conductivity, which was 3.4-fold (R = 0.81, *p* = 0.0001) and 10.8-fold (R = 0.99, *p* = 0.0001) higher than those detected at the start and end of the transect. Similarly, Cl^−^ levels were 4.8-fold (R = 0.53, *p* = 0.0001) and 10.7-fold (R = 0.64, *p* = 0.0001) higher in middle soils, respectively.

**Table 2:** ANOSIM comparing environmental parameters across Old Wallow.

Soil parameter		Start—Mid		Mid—End		Start—End	
		R	*p*-value	R	*p*-value	R	*p*-value
Geographical	MSL^a^	0.92	0.0001	0.79	0.0001	0.44	0.0001
	DMF	0.45	0.0001	0.46	0.0001	0.06	0.0176
	Conductivity	0.81	0.0001	0.99	0.0001	0.21	0.0002
	pH	0.18	0.0001	0.24	0.0001	0.15	0.0009
Macronutrients (mg/kg)	Total C	0.31	0.0001	0.36	0.0001	0.05	0.0267
	Total N	0.29	0.0001	0.15	0.0003	0.03	0.0834
	Total P	0.22	0.0001	0.12	0.0023	0.17	0.0001
Water extractable ions (ppm)	Cl^−^	0.53	0.0001	0.64	0.0001	0.07	0.0111
	NO_3_^2−^	0.18	0.0001	0.05	0.0163	0.16	0.0005
	PO_4_^3−^	0.21	0.0001	0.14	0.0004	−0.02	0.8031
	SO_4_^2−^	0.29	0.0001	0.82	0.0001	0.24	0.0001
Metal oxides (%)	SiO_2_	0.21	0.0001	0.02	0.1749	0.10	0.0009
	TiO_2_	0.30	0.0001	0.56	0.0001	0.08	0.0056
	Al_2_O_3_	0.36	0.0001	0.95	0.0001	0.17	0.0001
	Fe_2_O_3_	0.77	0.0001	0.63	0.0001	0.00	0.4239
	MnO	0.34	0.0001	0.15	0.0002	0.02	0.1237
	MgO	0.16	0.0001	0.14	0.0001	0.01	0.1878
	CaO	0.09	0.0007	0.46	0.0001	0.23	0.0001
	Na_2_O	0.73	0.0001	0.92	0.0001	0.01	0.1904
	K_2_O	0.26	0.0001	0.11	0.0016	0.18	0.0001
	P_2_O_5_	0.26	0.0001	0.05	0.0216	0.20	0.0001
	SO_3_	0.25	0.0001	0.77	0.0001	0.22	0.0001

a Mean sea level.

### Old Wallow harbours a high abundance of the novel taxa at the family level

**Figure 1:**
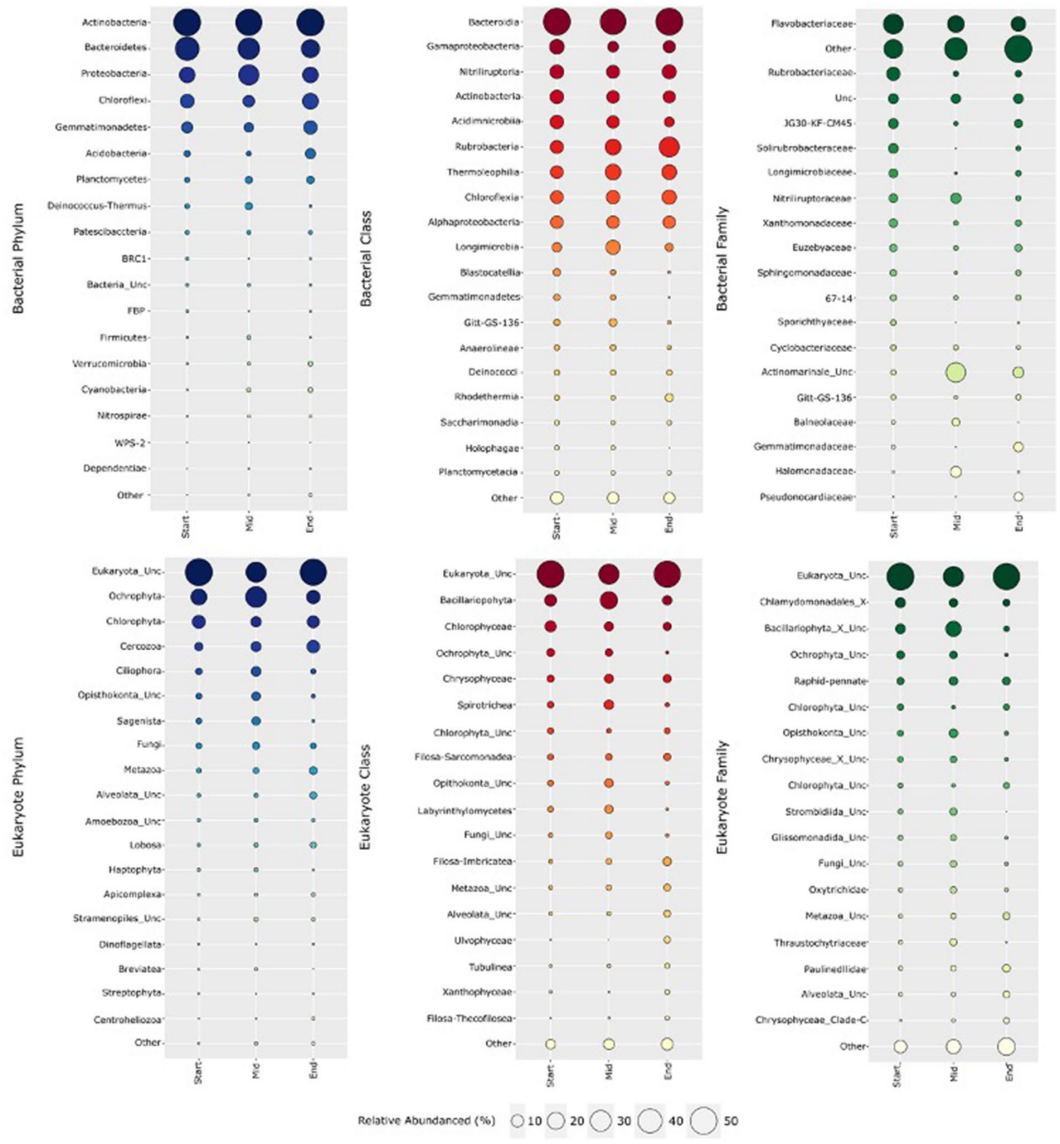
Relative abundances of bacteria (top) and eukarya (bottom) along the 300 m long OW transect at phylum, class and family level. *Actinobacteria* was the most dominant bacterial phyla, comprising *Rubrobacteria*, *Actinobacteria* and *Nitriliruptoria* at class level. At family level—*Ca.* Actinomarinales and *Halomonadaceae* were in greater abundances in the middle transect soils. Eukaryotes were largely unclassified. Dominant phyla included the photosynthetic algae *Ochrophyta* and *Chlorophyta*. Class and family level abundances were highly variable with unclassified *Bacillariophyta* (av 16%) and unclassified *Opisthokonta* (av 4.8%) dominating the lowest elevation, middle transect soils.

Amplicon sequencing of all 93 soil samples yielded a total of 9,896,141 bacterial SSU rRNA gene sequences and 3,777,586 eukaryotic gene sequences after read-quality filtering. A total of 10,864 bacterial zOTUs were clustered and classified into 91 classes spanning 31 phyla, with the number of zOTUs per sample ranging from 901 to 3,339, with the lowest numbers recovered from soils at the start of the transect (Supplementary material S3). Comparatively, 1,387 eukaryotic zOTUs were clustered and classified into 68 classes spanning 34 phyla, spanning from 47 to 439 zOTUs per sample, with lower numbers also recovered in soils at the beginning of the transect. Rarefaction curves of transect locations revealed that bacterial and eukaryotic richness approached asymptote, confirming that sample sequencing sufficiently represented their true microbial diversity (Supplementary Figure S3, Supplementary material S2). On average, across all 93 samples, bacterial zOTUs associated with *Actinobacteria* (36.8%), *Bacteroidetes* (22.8%), *Proteobacteria* (14.9%), *Chloroflexi* (9.8%) and *Gemmatimonadetes* (6.6%) were dominant, collectively accounting for 90.9% of the bacterial sequences recovered (Figure 1). Other common soil phyla represented a lower proportion of the Old Wallow microbiome, with *Acidobacteria*, *Planctomycetes* and *Deinococcota* accounting for an average 2.7, 2.0 and 1.1% of the bacterial communities, respectively (Supplementary material S3). Family level abundances were highly variable across the site, dominated by *Bacteroidia* (av 21.0%), *Acidimicrobiia* (av 14.2%) and *Gammaproteobacteria* (av 8.9%). *Rubrobacteria*, *Thermoleophilia* and *Longimicrobia* were in higher relative abundances at the start of the transect, while *Gammaproteobacteria*, *Acidimicrobiia*, BD2-11_terrestrial_group and *Rhodothermia* were in highest abundances in the middle soils, up to 15.2, 24.3, 1.3 and 4.0% of the bacterial community, respectively. At the family level, rare and uncultured taxa were dominant, with a high relative abundance of *Flavobacteriales* (17.9%), *Ca.* Actinomarinales (9.6%), and *Rubrobacterales* (4.8%) observed. Highest abundances of *Ca.* Actinomarinales (av 27.8%), *Nitriliruptoriaceae* (av 6.1%) and *Halomonadaceae* (av 6.8%) were observed in the middle, high Cl^−^ soils (Table 1, Supplementary material S3).

At phylum level, *Ochrophyta*, *Chlorophyta* and *Cercozoa* each represented 21.4, 9.3 and 7.2% of the eukaryotic communities at OW, respectively (Figure 1). At lower abundances (1–5%) were *Ciliophora* (3.8%), *Opisthokonta* (2.9%), *Sagenista* (2.6%), *Fungi* (2.6%), *Metazoa* (2.4%), *Alveolata* (1.5%) and *Lobosa* (1.2%) altogether accounting for a substantial portion of the eukaryotic communities (Supplementary material S3). High proportions of novel and under-characterised eukaryotic taxa were identified at OW, with 41.6% of zOTUs unclassified at the phylum level and a further 22 rare phyla (<1% abundances each) accounting for the remaining 3.7% of the population. While class level communities were predominantly unclassified (43.3%), *Bacillariophyta* (av 13.1%), *Chlorophyceae* (av 6.1%), *Chrysophyceae* (av 4.3%), *Spirotrichea* (3.3%), unclassified *Ochrophyta* (av 2.9%), and unclassified *Chlorophyta* (av 1.9%) were dominant, with *Bacillariophyta*, *Spirotrichea* and *Opisthokonta*_unclassified in higher abundances in the middle soils, alongside elevated soil moisture and nutrient content (Figure 1, Tables 1, 2). At the family level, novel or unclassified classes dominated the community (i.e. *Opisthokonta*_unclassified, *Ochrophyta*_unclassified, *Strombidiida*_unclassified and *Labyrinthulomycetes*_unclassified) (Figure 1, Supplementary material S3). Similar trends in terms of variability across the site was also observed, with *Bacillariophyta*_X_unclassified ranging between relative abundances of av. 1.9–16.1%, with significantly (*p* < 0.05) higher levels detected in middle soils.

### Alpha diversity measures indicated a high richness and diversity within OW soil

The diversity of bacteria and eukarya was analysed along the three transect groups using four diversity indices: Simpson, Shannon, Richness, and Evenness. Overall, diversity indices were greater for bacteria (Simpson = 0.98, Shannon = 5.31, Richness = 1929.38) than eukarya (Simpson = 0.91, Shannon = 3.52, Richness = 205.41) (Table 3). However, evenness was similar between the two domains, with an overall value of 0.70 for bacteria and 0.67 for eukarya. The Simpson index ranged from 0.97 to 0.99 for bacteria, with the highest values observed in the end soils. Shannon and Richness exhibited the same trends, ranging from 5.01 to 5.89 for Shannon and 1597.88 to 2527.43 for Richness. Evenness ranged from 0.675 to 0.75, with no significant differences observed between transect groups. The Simpson index ranged from 0.86 to 0.94 for eukarya, with the highest diversity observed in the mid-group soils (Table 3). Shannon and Richness were also highest in middle soils, which were highest in moisture (Table 1, Supplementary Figure S4), ranging from 3.09 to 3.84, and 146.21 to 264.33, respectively (Table 3). The Evenness index ranged from 0.63 to 0.69, with no significant differences observed between transect groups.

**Table 3:** Alpha diversity measures at Old Wallow.

	Transect section^a^	Simpson	Std Dev	Shannon	Std Dev	Richness	Std Dev	Evenness	Std Dev
Bacteria	Total	0.98	0.012	5.31	0.59	1929.38	616.98	0.70	0.052
	Start	0.98	0.013	5.06	0.453	1597.88	460.17	0.69	0.042
	Middle	0.97	0.01	5.01	0.45	1695.97	394.29	0.675	0.0425
	End	0.99	0.0059	5.89	0.41	2527.43	516.80	0.75	0.035
Eukarya	Total	0.91	0.082	3.52	0.64	205.41	86.62	0.67	0.098
	Start	0.86	0.12	3.09	0.79	146.21	78.39	0.63	0.14
	Middle	0.94	0.022	3.84	0.29	264.33	61.55	0.69	0.047
	End	0.93	0.037	3.67	0.41	211.6	74.92	0.69	0.067

a For each transect section *n* = 31 and Total = 93 soil samples.

Spatial variation in bacterial relative abundances was also observed for transects groups (Figure 1). ANCOM-BC analysis revealed significant differences (*p* < 0.05) between 11 different bacterial phyla across the three soil groupings when filtered for relative abundances above 0.5% (Supplementary material S2). The largest shifts in relative abundances were observed in *Deinococcus-Thermus,* between soils in the mid and end transect groups (log fold change = 2.73, *p* = 3.86 × 10^−44^) and between the start and middle groups (log fold change = 2.14, *p* = 7.41 × 10^−25^). A significant (*p* < 0.05) shift in *Acidobacteria* relative abundance was observed between the start and end groups (log fold change = 2.11, *p* = 6.97 × 10^−9^) and between the mid and end groups (log fold change = 1.43, *p* = 1.29 × 10^−36^), with no significant change observed between the start and mid soil group. *Actinobacteria* exhibited minor shifts in relative abundances between the start and mid soils (log change = 0.826, *p* = 1.99 × 10^−36^), as well as between the start and the end of the transects (log change = 0.366, *p* = 3.48 × 10^−4^) (Supplementary material S2).

ANCOM-BC analysis of eukaryotes revealed that 15 different phyla exhibited significant shifts (*p* < 0.05) in community composition across the three transect groups (Supplementary material S2 and Figure 1). The phylum *Ciliophora* exhibited the largest shift in abundance between the start and mid groups (log fold change = 2.47, *p* = 9.66 × 10^−5^) and between the start and end soils (log fold change = −2.25, *p* = 0.008). *Cercozoa* also showed significant changes between the start and end of the OW transect (log fold change = 2.26, *p* = 2.17 × 10^−10^). In addition to these phyla, other significant differences in abundance were observed, including *Alveolata*_unclassified (log fold change = 1.44, *p* = 0.0169) and *Amoebozoa*_unclassified (log fold change = 1.70, *p* = 0.0029) between the mid and end groups, and *Apicomplexa* (log fold change = 1.48, *p* = 0.0044) between the mid and end groups. *Lobosa* showed significant differences between all three groups, with the largest shift in abundance observed between the mid and end soils (log fold change = 2.06, *p* = 1.09 × 10^−12^). *Metazoa* also exhibited significant changes between all three groups, with the largest shift in abundance observed between the mid and end of the transect (log fold change = 1.88, *p* = 0.0041).

### Interlinked communities with *Actinobacteria* forming the backbone of the network

**Figure 2:**
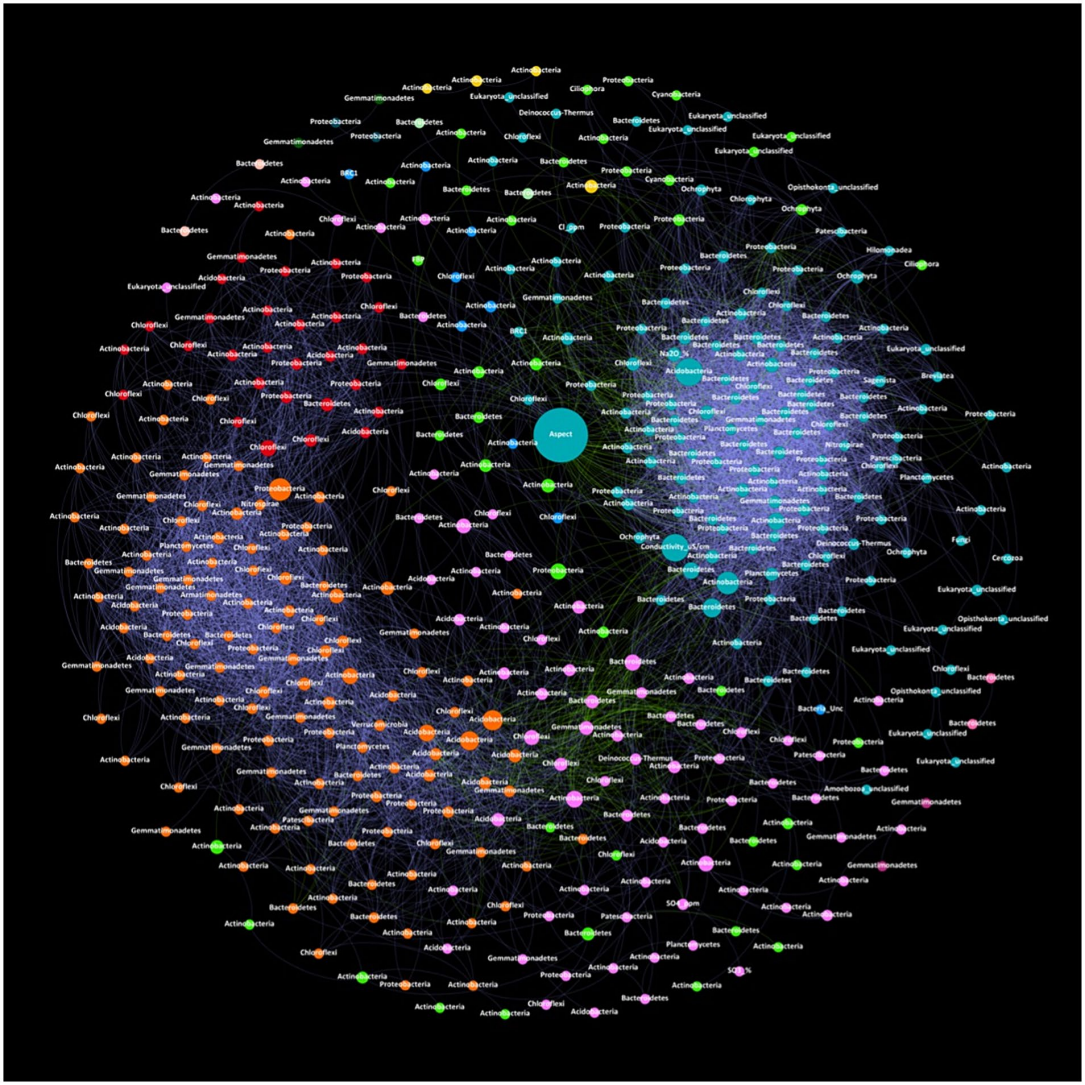
Co-occurrence network of correlations between the relative abundance of zOTUs and soil physiochemical parameters within OW. Correlations between zOTU pairs were calculated using the maximal information coefficient (MICe). Results were visualised in Gephi with node size associated with betweenness centrality and node colour with network modularity. Edge colour is associated with negative (green), or positive (purple) correlations based on Pearson coefficients. *Actinobacteria* accounted for the highest number of nodes thus forming the backbone of the network. Aspect and conductivity have high betweenness centrality suggesting a high influence over community composition, illustrated by a high number of positive edges associated linked with a large cluster (aqua) of *Acidobacteria*, *Actinobacteria* and *Bacteroidetes*.

Phylum-level networks displaying the co-occurrence of zOTUs and environmental parameters provided insights into the drivers of bacterial and eukaryotic relationships and interactions (Figure 2). The resulting network consisted of 425 nodes (clustering coefficient = 0.657) with 7,149 edges across six connected components with a network diameter of eight edges (Supplementary material S4). Of the 425 nodes, bacterial phyla accounted for 390 nodes, eukaryotes accounted for 29 nodes, and various environmental parameters also accounting for nodes including aspect, Cl^−^, conductivity, Na_2_O, SO_3_ and SO_4_^2−^. The microbial network in Old Wallow exhibited a highly interlinked community with 7,148 significant (MICe >0.8, *p* < 0.05) connections between zOTUs (Figure 2). The network consisted of 13 communities, with the three largest consisting of 138, 120, and 73 nodes, respectively. The highest node abundance was observed for phyla *Actinobacteria* (nodes = 134), followed by *Bacteroidetes* (nodes = 75), *Chloroflexi* (nodes = 54), *Proteobacteria* (nodes = 50) and *Gemmatimonadetes* (nodes = 32). At the class level *Gillisia* had the highest number of nodes (nodes = 43) followed by *Gemmatimonadaceae*_Unc (nodes = 21), *Actinomarinales*_Unc (nodes = 16) and *Rubrobacter* (nodes = 13) (Supplementary material S4). Betweenness centrality represents the number of shortest paths going through a node. Nodes with high betweenness centrality included Aspect, Conductivity, *Nitriliruptoraceae* (zOTU662), *Subgroup-7* (zOTU328), *Balneolaceae* (zOTU535) and *Alphaproteobacteria* (zOTU1520). Network statistics analysed included modularity with a value of 0.502, the number of communities (n = 13), and the average clustering coefficient (a measure of how closely nodes in a graph cluster together) with a value of 0.657 (Supplementary material S4). There was a total of 6 connected components (where all nodes are reachable from each other), the graph density (which represents the ratio of the edges present in a graph to the maximum number of edges that the graph can contain) had a value of 0.079, and the diameter (which is the length of the shortest path between the most distant nodes) had a value of 8.

### Microbial communities respond variably to soil environmental gradients

**Figure 3:**
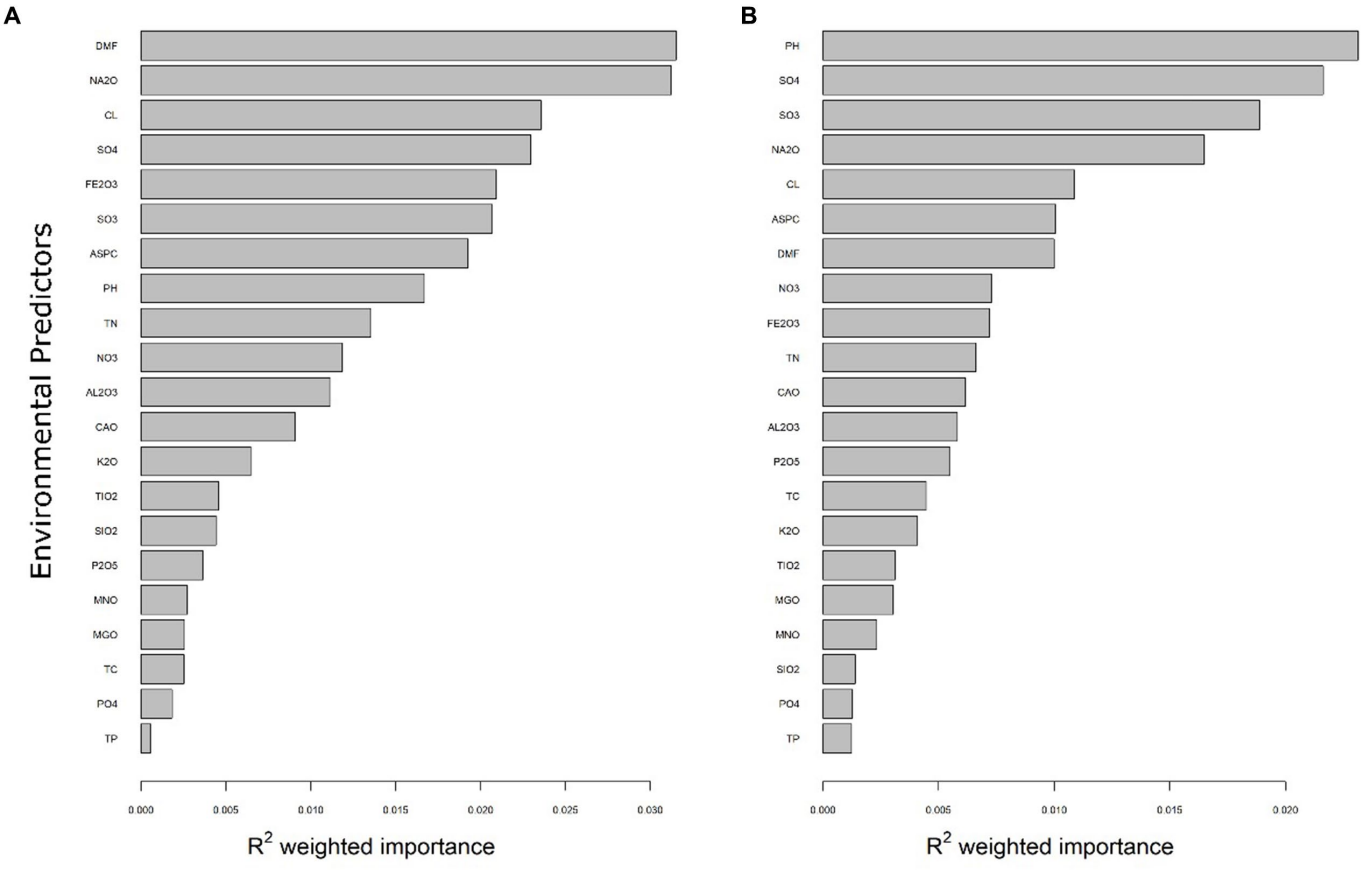
Environmental variables correlating to the OW soil communities. R2 weighted importance of predictors driving distributions of soil bacteria **(A)** and eukarya **(B)** within OW. The environmental variables analysed predicted a moderate fraction of soil community distributions. Rank-order importance differed between domains, with DMF (R^2^ = 0.035), Na_2_O (R^2^ = 0.030) and Cl^−^ (R^2^ = 0.024) the most robust predictors for bacteria, while pH (R^2^ = 0.020), SO_4_^2−^ (R^2^ = 0.025) and SO_3_ (R^2^ = 0.019) and were strongest predictors of the eukaryotic communities.

Gradient Forest (GF) analyses were implemented to identify robust predictors that correlated with shifts in bacterial and eukaryotic community composition across Old Wallow. Overall importance plots (R^2^ weighted values) were used to show the rank-order importance of 26 significant environmental predictors as determined by the GF model (Figure 3, Supplementary Table S1). Gradient Forest analyses showed that the most robust predictors for both the bacterial and eukaryotic communities were SO_3_, SO_4_^2−^, Na_2_O and DMF. For bacteria, DMF (R^2^ = 0.035) and Na_2_O (R^2^ = 0.030) were most strongly correlated, while pH (R^2^ = 0.026) was the most robust eukaryotic predictor. Additionally, SO_4_^2−^ was a strong predictor for both the bacterial (R^2^ = 0.023) and eukaryotic communities (R^2^ = 0.025). Spearman analysis was conducted to assess the significance and direction of these environmental correlations (Supplementary Figure S5). At the phylum level, a positive correlation was observed between *Acidobacteria* and PO_4_^3−^, exhibiting a Spearman correlation coefficient of 0.39. Conversely, *Acidobacteria* demonstrated strong negative correlations with both SO_4_^2−^ and SO_3_, with Spearman correlation coefficients of −0.78 and −0.74, respectively. Exploring correlations at the family level revealed significant findings, with a strong positive correlation between *Halomonadaceae* and both conductivity and sodium oxide, each with a Spearman correlation coefficient of 0.79. Similarly, *Nitriliruptoraceae* was positively correlated with conductivity and SO_4_, with Spearman coefficients of 0.79 and 0.76, respectively. On the other hand, notable negative correlations were observed between *Solirubrobacteraceae* and Na_2_O (Spearman = −0.78), and between *Gemmatimonadaceae* and conductivity (Spearman = −0.77).

**Figure 4:**
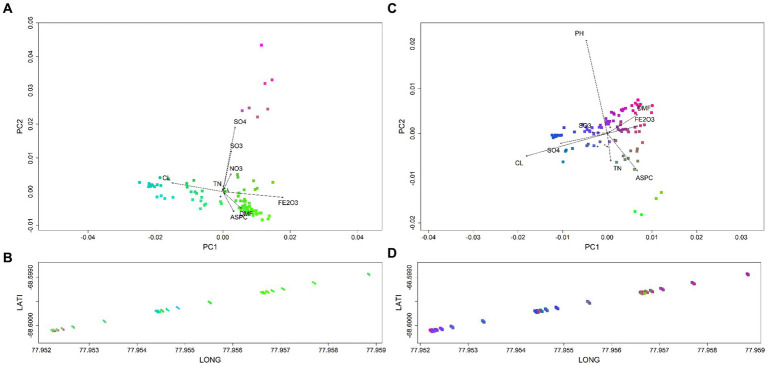
Biological and geographical biplots classifying spatial groups for polar soil bacteria **(A,B)** and eukaryote communities **(C,D)** across OW. Different colours highlight spatial compositional groups and associated shifts across the transect. Key drivers of bacterial groups observed at the start and end of the transects were DMF, Fe_2_O_3_ and ASPC. While Cl^−^ was the key driver of bacterial communities in the middle cluster of the transect. A distinct group at the start of the transect was predominantly driven by SO_4_^2−^ and SO_3_. Eukaryotic spatial groups were less distinct but had similar drivers as bacteria based on transect location. Communities at the start and end were strongly correlated to DMF and Fe_2_O_3_, with mid cluster compositional groups also driven by SO_3_, SO_4_^2−^ and Cl^−^.

Biological and geographical biplots allowed for the classification of microbial spatial groups across OW, with colour variation highlighting shifts in species composition and turnover (Figure 4). Key drivers that led to the compositional turnover of bacterial groups at the start and end of the transect were DMF, Fe_2_O_3_ and Aspect (Figure 4, Supplementary Figure S5). For communities in the mid-group of the transect, Na_2_O and Cl^−^ were the key drivers. Soil communities at the start of the transect were distinct, with both the bacteria and eukaryotes predominantly correlating with SO_4_^2−^ and SO_3,_ with eukaryotes also correlating with NO_3_^−^ (Figure 4). Eukaryotic spatial groups were less distinct but had similar drivers as bacteria based on transect location (Figures 4C,D). For example, eukaryotic communities in the middle soils, at the lowest elevation point (Supplementary Figure S2), were associated with Na_2_O, Cl^−^ and pH (Figures 2, 4). In contrast, communities in soils at the end of the transect correlated with DMF and Aspect.

### Predicted thresholds or environmental tipping points along Old Wallow

**Figure 5:**
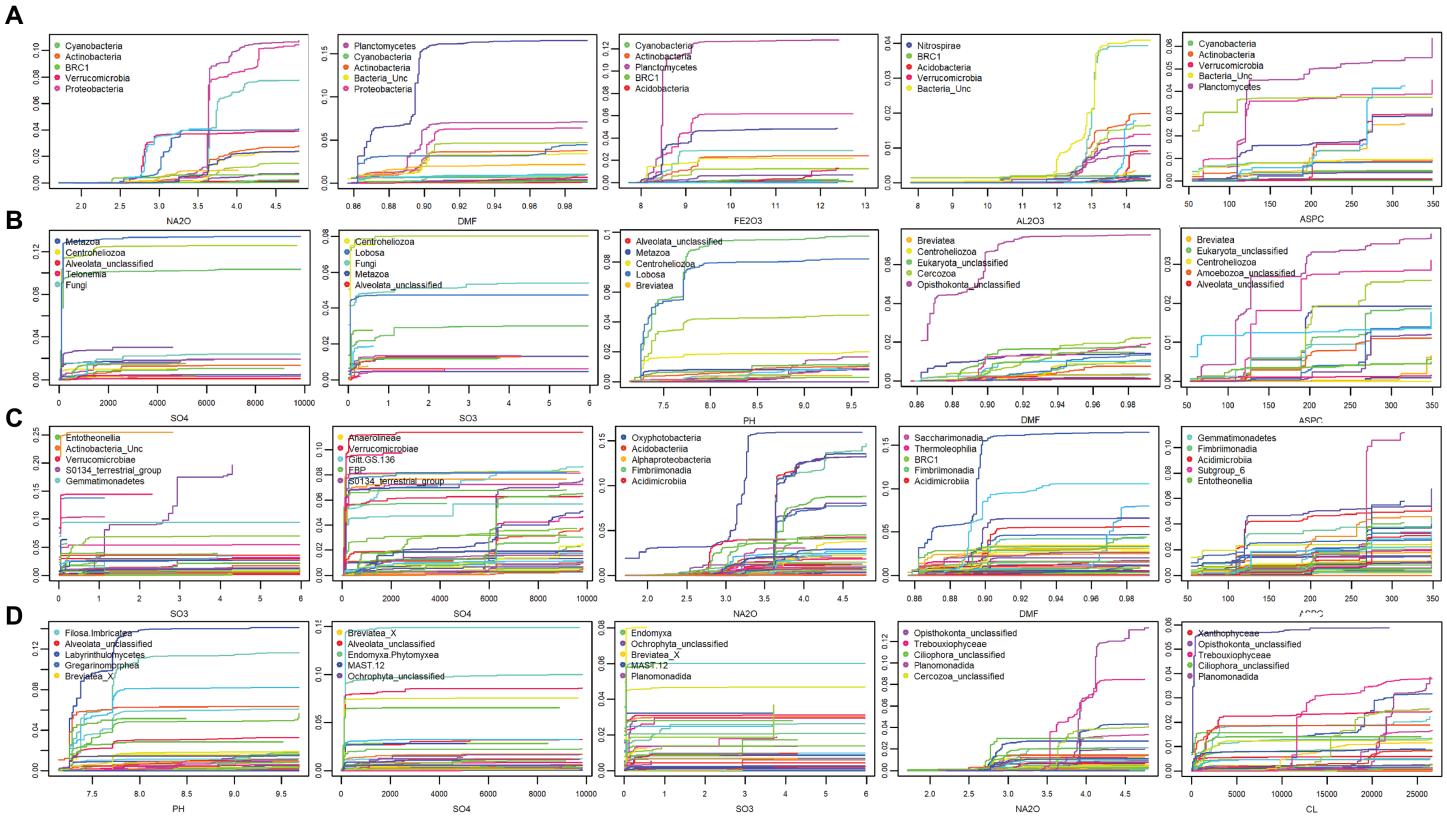
Cumulative plots that visualise compositional turnover or “splits” along environmental gradients at OW with the top five predictors shown for phylum level **(A)** bacteria and **(B)** eukaryotes **(C)** Class level for bacteria **(D)** eukaryotes. The y-axis displays cumulative importance on the most responsive taxa, with each coloured line representing a different phylum. Steep slopes or “splits” indicate high compositional turnover at that environmental threshold. Splits along edaphic gradients were generally non-uniform highlighting variable rates of change in phylum abundances. At class level, the top predictors were SO_3_, SO_4_^2−^, Na_2_O for both domains. The most responsive classes were largely unclassified taxa. Notable splits were observed at 3.7% Na_2_O for *Proteobacteria,* **(C)** *Planctomycetes, Cyanobacteria* **(A)**, Oxyproteobacteria, Acidimicrobiia and S0134_Terrestrial group **(C)** and at 100 ppm SO_4_^2−^_,_ for *Lobosa*, *Cercozoa*, and Eukaryota_unclassified **(B)** and Breviatea_unc, Endomyxa.Phytomyxra, Alveolata_unc **(D)**.

Predictor cumulative plots were used to visualise “splits” or cumulative change in the composition of the microbial community along the environmental gradients, enabling the identification of critical thresholds or tipping points for microbial composition turnover (Figure 5, Supplementary Figures S6, S7). Splits in community composition along soil environmental gradients were generally non-uniform, indicating variable rates of change in species abundances. For bacteria at phylum level, the highest rates of compositional turnover, as indicated by a spike in accuracy importance (Figure 5A) and steep splits on the cumulative plots were observed for *Phycisphaerae* (blue line) at 3% Na_2_O, while *Proteobacteria*, *Acidobacteria* (red line), and *Planctomycetacia* (purple line) are predicted to shift in abundance at 3.7% Na_2_O (Figure 4A). Notable splits in community composition were also exhibited for DMF at 0.89 (0.11% moisture) for *Actinobacteria* and *Proteobacteria*. A notable split at 8.5% Fe_2_O_3_ was observed for *Planctomycetacia* (purple line), while two thresholds were observed for Al_2_O_3_; at 12.75% (*Rhodothermia-aqua* line) and 13.75% (*Subgroup-6*—light blue line). Aspect was also a strong predictor, with three compositional turning points predicted at 125 (Southeast), 200 (Southwest), and 260 (West). Analysis of the top eight predictors plotted against soil bacterial communities along the transect showed that indeed, soils in the middle of the transect, that were high in moisture, Na_2_O, Cl^−^ and Fe_2_O_3_ exceeded the predicted thresholds (Figure 4). At the family level, the top environmental predictors for bacteria were SO_3_, SO_4_^2−^, Na_2_O, DMF, ASPC, and Cl^−^ with *Oxyphotobacteria*, and uncultivated taxa (e.g., Gitt.GS.136, S0134_terrestrial group, PAUC43f_marine_benthic_groupTK10) most responsive (Figure 5, Supplementary Figure S6). These predicted community shifts coincided with increases in the relative abundances of *Ca.* Actinomarinales (av 27.8%), *Nitriliruptoriaceae* (av 6.1%) and *Halomonadaceae* (av 6.8%) (Figure 1, Supplementary material S3).

For eukaryotes, SO_4_^2−^ was a strong predictor, with one critical threshold at approximately 100 ppm observed, with three phyla responding, namely *Lobosa* (light blue line) *Cercozoa* (green line) and Unclassified*-*Eukaryotes (light green line) (Figure 5B). Additionally, 0.02% SO_3_ strongly predicted *Lobosa, Centroheliozoa*, and *Fungi*. For pH, a steep split was observed at pH 7.75, for *Lobosa*, Unclassified-Eukarya (aqua line), and *Cercozoa* (green line). While DMF led to two tipping points, with turnover at 0.86 and 0.90% DMF predicted, *Opsithokonta* responded most strongly in this case. Aspect also correlated to three critical thresholds for eukaryotes, with compositional turnover observed at 125 (Southeast), 200 (Southwest), and 260 (West). At the family level, the top predictors were SO_3_, SO_4_^2−^, and Na_2_O for both domains, with major splits observed for bacteria at DMF 0.89% (Gitt.GS.136, *Oxyphotobacteria*), and for eukaryotes at both 100 ppm (*Opisthokonta*_unclassified) and 11,000 ppm Cl^−^ (*Trebouxiophyceae*) (Figure 5, Supplementary Figure S7). The most responsive families were largely unclassified taxa (eg *Opisthonkta*_unclassified, *Alveoata*_unclassified, *Ochrophyta*_unclassified, *Breviatea*_X, MAST12), with major thresholds predicted for *Opisthonkta*_unc and MAST12 at 3.9% Na_2_O, and for *Filosa.Imbricatea*, *Cercozoa*_unc, and *Alveoata*_unc at 100 ppm SO_4_^2−^ (Supplementary Figure S7).

## Discussion

The Old Wallow ecosystem includes the presence of meltwater and stochastic nutrient input from local wildlife (Seppelt and Broady, 1998; Zhang et al., 2020), sharing many environmental constraints with the greater Vestfold Hills, including low moisture and nutrient scarcity (Zhang et al., 2020). But, unlike other sites in this region, namely Adams Flat, Rookery Lake and Heidemann Valley, clear soil chemical gradients were identified that are likely due to the nearby presence of an elephant seal wallow, with on average, >2-fold higher concentrations of total N, total C, calcium, sulphates, sodium and conductivity (Table 1) detected (Zhang et al., 2020; Zhang et al., 2024). Such elevated levels of nutrient inputs to soils are reported to occur, not only from sea spray, given the location of OW the coast, but also from remnants of seal wallows (Ball et al., 2015; Guo et al., 2018). Alongside elevated nutrient levels, microbial communities appeared to be impacted at lower taxonomic levels, with a widespread dominance of rare, candidate bacteria (*Ca.* Actinomarinales, Gitt-GS-136), and unclassified eukaryotes (*Bacillariophyceae*_unc, *Ochrophyta*_unc), many of which have been exclusively identified as marine taxa thus far (Bowman et al., 2000; López-Pérez et al., 2020; Li et al., 2023). In cold regions, the slow degradation of seal carcasses and accumulated seal aggregate provides gradual macromolecular input into the surrounding soil over an extended period (Zvěřina et al., 2017). We found elevated levels of nutrients in the soils at the start of the sampling transect, closest to the wallow, possessing significantly (*p* < 0.05) higher levels of total C, total P, sulphate, sulphide, and nitrate (Tables 1, 2). This phenomenon has been documented at other Antarctic sites, including northern James Ross Island (Zvěřina et al., 2017), the McMurdo Dry Valleys (Tiao et al., 2012), Maritime Antarctica and Marion Island (Dai et al., 2020), and can be attributed to the combination of moulting skin, urination and defaecation by local penguin and elephant seal populations (Panagis, 1985).

The transfer of marine-derived nutrients into terrestrial ecosystems in the sub-Antarctic and Antarctica are known to be strong drivers of soil communities in cold regions (Zwolicki et al., 2015). Wind-blown fertilisation by marine-derived nutrients can occur over distances of several 100 m from vertebrate concentrations, such as penguin colonies and seal wallows, resulting in positive drivers of Antarctic terrestrial biodiversity (Bokhorst et al., 2019; Almela et al., 2022). In addition to a measurable macromolecular gradient, soils in the middle of the Old Wallow sampling site contained elevated levels of moisture, Cl^−^, Na_2_O, MgO and conductivity, with the accumulation of salts in the middle of the transect likely due to their position at the lowest elevation of the site (Table 1, Supplementary Figure S2). In coastal Antarctic regions, marine-derived aerosols containing high salt loads are common (Hall and Wolff, 1998; Hara et al., 2006). Therefore, salt accumulation in Old Wallow soil could be explained by the site’s close coastal proximity and North-West aspect, resulting in moisture and salt input from sea spray exposure. Furthermore, the lowest elevation soils were positioned in the middle of the transects (Supplementary Figures S2, S4), possibly resulting in the flow and accumulation of moisture and soluble salts to this location, with the latter increasing further due to evapo-concentration. This is consistent with soils from the McMurdo Dry Valley, where Cl^−^ originating from sea spray or meltwater has accumulated. Lower elevation sites have also been strongly correlated with higher Cl^−^ concentrations in the Shackleton Glacier region, despite a strong positive correlation between elevation and total salt content (Diaz et al., 2021).

The Vestfold Hills is considered an Antarctic oasis or refuge harbouring a diverse microflora community (Line, 1988). At the phylum level, the bacterial community composition of Old Wallow closely resembles that of terrestrial Antarctica^17^, particularly sites within the Vestfold Hills (Cary et al., 2010; Zhang et al., 2020; Varliero et al., 2024), with soils dominated by *Actinobacteria* (36.8%), *Bacteroidetes* (22.8%), and *Proteobacteria* (14.8%) (Figure 1). Co-occurrence network analysis highlighted a complex microbial community, evidenced by more than seven thousand connections among 425 distinct entities (Figure 2). Such as highly connected network, combined with strong correlations with environmental variables, indicates a highly connected community structure, with organisms driving each other and reacting similarly to environmental drivers (Ferrari et al., 2016). In this ecosystem, the widespread diversity and connectedness of *Actinobacteria* suggests an interdependency on *Actinobacteria* within the Old Wallow ecosystem. The ubiquity of *Actinobacteria* is well established, with the metabolic versatility of this phylum enabling it to adapt and thrive in extreme environmental conditions (Lewin et al., 2016; Ji et al., 2017). Atmospheric chemosynthesis, the ability to oxidise trace gases to perform carbon fixation, is widespread in soils in the Vestfold Hills, with *Actinobacteria* implicated as the dominant bacterial phyla supporting primary production in this region alongside photosynthesis (Ji et al., 2017; Ray et al., 2022).

Similar to previous reports of Antarctic microbial dark matter (Lewin et al., 2016; Bowman, 2018), below phylum level diversity comprised a high proportion of unclassified and uncultured taxa. Most striking was an unusually high dominance of marine and halotolerant *Ca.* Actinomarinales (av 27.8%), *Nitriliruptoriaceae* (av 6.1%) and *Halomonadaceae* (av 6.8%) in middle transect soils that were high in conductivity, moisture and chlorine (Figure 1). This is unique as *Ca.* Actinomarinales have been exclusively reported in marine environments (López-Pérez et al., 2020). In contrast, the highest abundances of JG30-KF-CM45 (av 5.6%) and *Rubrobacteriaceae* (av 10.6%) occurred in the soils closest to the wallow, alongside elevated nutrients and salts. Such high levels of halophiles can be attributed to the Vestfold Hills being comprised of >34 chemically stratified saline lakes and marine basins, that formed over 10,000 years ago via isostatic glacial marine uplift (Gibson, 1999; Bowman et al., 2000). *Ca.* Actinomarinales has no cultured representatives, and knowledge about their contributions to soil ecosystems is limited. However, metagenome-assembled genomes (MAGs) have shown their possession of small, streamlined genomes optimised for oligotrophic niches (López-Pérez et al., 2020). Furthermore, heliorhodopsin genes have been detected, implicating this family as potential photoheterotrophs and important facilitators of carbon mineralisation (Ghai et al., 2013). It is recommended that OW soils are thus used as a natural enrichment source for the isolation and characterisation of these uncultured taxa due to their high relative abundances and their potential importance to soil microbiome functions.

Chemoheterotrophic and halotolerant psychrophilic bacteria, including *Flavobacteria*, are known to be more abundant in coarse, sandy soils with high Cl^−^ concentrations (Oren, 2002; Müller and Oren, 2003), a characteristic prevalent in OW soils (Table 1). This abundance aligns with their mechanism of using chloride to osmotically balance their cytoplasm with the surrounding environment. *Flavobacteria* play a vital role in ecological processes, particularly in the uptake and degradation of complex organic matter (Buchan et al., 2014; Almela et al., 2022), which is crucial for nutrient cycling within the greater OW microbial network. Our co-occurrence network analysis further highlights the dynamic inter-taxa relationships in this ecosystem. Specifically, *Flavobacteriacea* exhibits a symbiotic relationship with *Ochrophyta*, accounting for 25% of algae-related interactions. This interaction is consistent with *Flavobacteria* forming commensal aggregates with diatoms, a relationship that likely benefits from the increased availability of organic matter in these environments (Grossart et al., 2005; Unnithan et al., 2013; Almela et al., 2022). In contrast, *Gillisia* demonstrates an interesting pattern within the network, forming the most connections (852) within the network but predominantly within its own group. This pattern suggests a preference for intra-species interaction, possibly minimizing its competitive interactions with other microbial populations (Unnithan et al., 2013; Wang et al., 2020).

In addition to novel bacterial taxa, OW soils are also inhabited by a high diversity of eukaryotes—and like other Antarctic sites in the region, the phyla *Ochrophyta* (21.4%) and *Chlorophyta* (9.3%) dominate (Zhang et al., 2024). While low abundances of *Cyanobacteria* were found, photosynthesis is likely a significant contributor to primary production in OW given the higher abundances of diverse phototrophic algae (e.g., *Bacillariophyta* av. 13.1%, *Chlorophyceae* av. 6%, and *Chrysophyceae* av. 4.4%) present (Figure 1). This high algal diversity suggests a complex and specialized ecosystem. However, the eukaryotic community was predominantly composed of unclassified lineages, accounting for 41.6% of its makeup. This was despite our utilization of the PR2 database, which is known for its more precise taxonomic classification of eukaryotes compared to other available options (Guillou et al., 2013). The high proportion of unclassifiable taxa within soil environments is common in studies that utilise the 18S rRNA gene for taxonomic classification (Gokul et al., 2013; Delgado-Baquerizo et al., 2018). This typically results from the low number of metabarcoding studies focusing on eukaryotic populations, reducing the effectiveness of the databases available (Guillou et al., 2013). Together with these findings, our study emphasises the need to uncover unclassified eukarya and their ecological role through novel cultivation techniques and extensive metagenomic sequencing of Antarctic soil environments.

Water availability (DMF-1) emerged as the most influential factor in shaping bacterial diversity, followed by sodium-oxide and chlorine (Figure 3, Supplementary Figure S5), with strong correlations widely reported between water availability and microbial richness and diversity (Barrett et al., 2007; Angel et al., 2010; Siciliano et al., 2014). Protist communities were predominantly marine and coastal species of protozoa, amoeba, and micro-algae, with predicted tipping points of 100 ppm Cl^−^, pH 7.75 and 3.7% Na_2_O (Figure 5). These tipping points correlated with shifts in the communities in the wetter, middle transect soils, towards photosynthetic algae (*Ochrophyta*) and uncultured protists (*Sagenista*), at the expense of *Metazoa* and Eukarya_unclassified (Figure 4, Supplementary Figures S6, S7). At the family level, a notable distinction of this community was an increase in relative abundances of unclassified *Bacillariophyceae* (diatoms), alongside increased bacterial diversity (Figure 1, Supplementary material S2).

Diatoms are unicellular algae that contribute significantly to carbon fixation, being reportedly responsible for 20% of global net primary production (Sethi et al., 2020). Micro-algae engage not only in photosynthesis but are metabolically flexible and capable of sulphate assimilation and reduction, playing crucial roles in carbon, nitrogen and sulphur cycling (Stefels et al., 2007; Galí Tàpias et al., 2015). Relationships between diatom community structures with conductivity, moisture and nutrient content here (Supplementary Figure S5), have been previously reported in diverse ecosystems (Saunders et al., 2009; Sethi et al., 2020). Such sensitivity to change is consistent with the application of diatoms as bioindicators during environmental assessments, for the detection of pollution, eutrophication and the success of habitat restoration (Blanco, 2024). Our study also found a positive relationship between Fe_2_O_3_ and bacterial phyla *Proteobacteria* and *Planctomycetes*, with the highest abundances occurring in the driest soils from the start of the transect, which were high in sulphates and Fe_2_O_3_ (Table 1, Figure 3). Hematite is known to act as an electron conduit, supporting respiration and growth in soil bacterial communities (Kato et al., 2010; He et al., 2011). Our findings align with earlier studies indicating enhanced abundances of these taxa with hematite addition (Afzal and Singh, 2022), marking a potential new understanding of its role in East Antarctic soils.

Given its proximity to an elephant seal wallow, Old Wallow is terrestrial ecosystem comprised of elevated soil nutrients that drive microbial community compositions. Soils harbour a diverse microbial community dominated by novel bacteria and eukaryotes that are yet to be characterised. To elucidate the ecological roles of uncultivated microbial groups and their contributions to nutrient cycling and ecosystem functioning, stable isotope probing, metagenomics and transcriptomics should be performed under native conditions (Starr Evan et al., 2021; Dang et al., 2023). We suggest that the edaphic thresholds identified here, combined with genome-resolved metagenomics be used to inform novel cultivation approaches for the isolation of these yet-to-be cultivated groups of bacteria and micro-algae. This study uncovers a deeper understanding of abiotic and biotic interactions at Old Wallow, uncovering tipping points around salts, moisture, and sulphates. This knowledge will be crucial for informing conservation and management efforts and maintaining the delicate balance of Antarctic ecosystems in the face of global environmental change.

## Data Availability

The datasets presented in this study can be found in online repositories. The names of the repository/repositories and accession number(s) can be found at: https://data.bioplatforms.com/organization/about/australian-microbiome, Australian-microbiome.

## Footnotes

^1^https://github.com/lh3/seqtk
