# Supplementary material for: The Vestfold Hills are alive: characterising microbial and environmental dynamics in Old Wallow, eastern Antarctica
Source: Front Microbiol. 2024 Sep 23;15:1443491. doi: 10.3389/fmicb.2024.1443491 (PMC11457671; doi:10.3389/fmicb.2024.1443491)
Supplement: Supplementary file 1 [file Data_Sheet_1.zip › Supplementary Figures, Table.PDF]

Supplementary Figures and Tables

Figure S1. Maps of the Old Wallow (OW) sampling site within the Vestfold Hills.

(A) The location of OW in the Vestfold Hills on the eastern Antarctic coast. (B) The geospatial transect sampling design used across OW and (B1) its location being 715m from a well-established elephant seal wallow. (B2) soil samples (n = 93) were taken at the following distance points along 3 parallel transects- 0, 0.1, 0.2, 0.5, 1, 2, 5, 10, 20, 50, 100, 100.1, 100.2, 100.5, 101, 102, 105, 110, 120, 150, 200, 200.1, 200.2, 200.5, 201, 202, 205, 210, 220, 250 and 300 m. (B3) Transects were sampled in triplicate (T1,T2,T3) with each parallel transect located three meters apart.

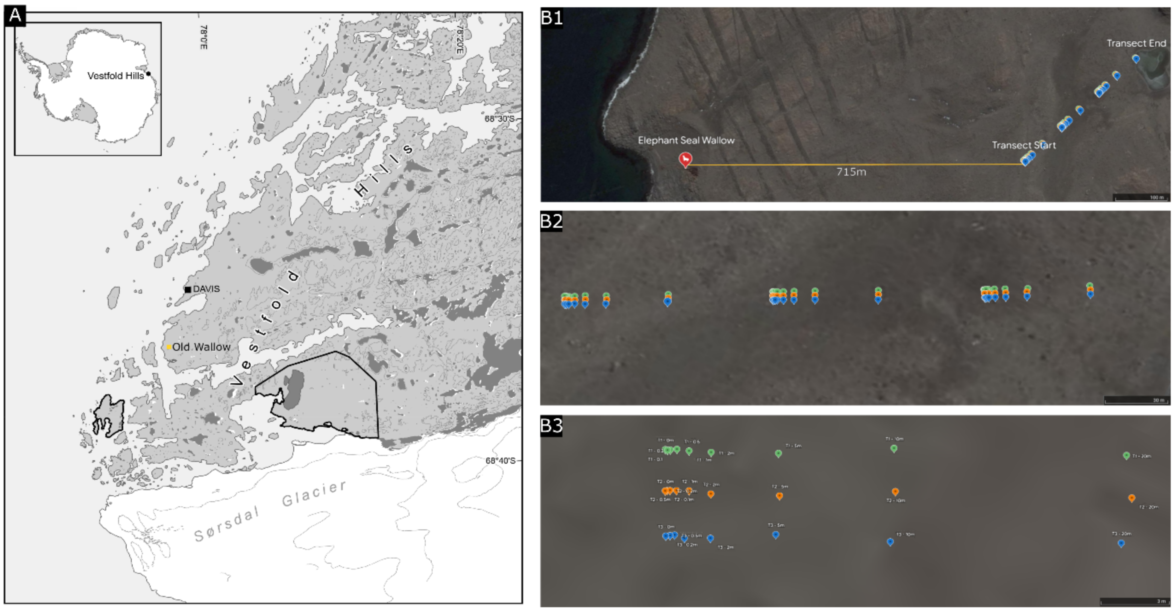

Figure S2. A 3D Scatterplot of elevation, along the OW transect. The elevation data (meters), relative to Mean Sea Level (MSL), plotted against the longitude and latitude of each sample was used to create this 3D scatterplot. Red represents samples from parallel transect line 1, green represents samples from parallel transect line 2 and blue represents samples from parallel transect line 3. The transects start at an elevation of 18 meters, dips to 15.5 meters at its midpoint, and then gradually rises to 18.63 meters towards the end, forming a subtle valley shape along the transect.

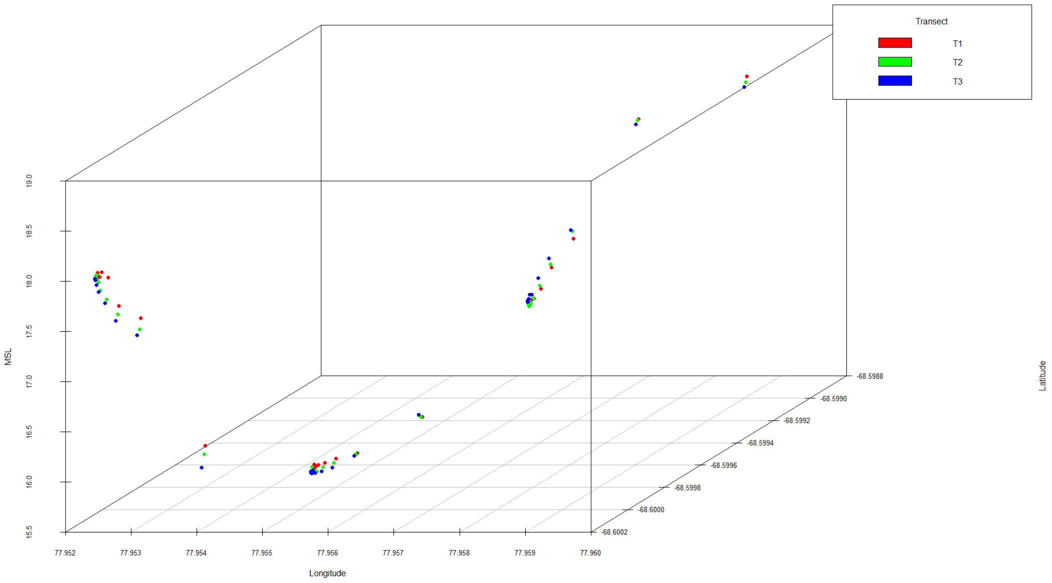

Figure S3. Rarefaction curves of subsampled bacterial and eukaryotic soil communities grouped into distances along the 300 m OW transect (i.e., start = 0-50, middle = 100-150, end = 200-300). In all cases, asymptote was reached indicating that sufficient sampling depth had been achieved. Soil grouped at the ‘End’ of the 3 - parallel transects contained the highest species diversity for both domains analysed.

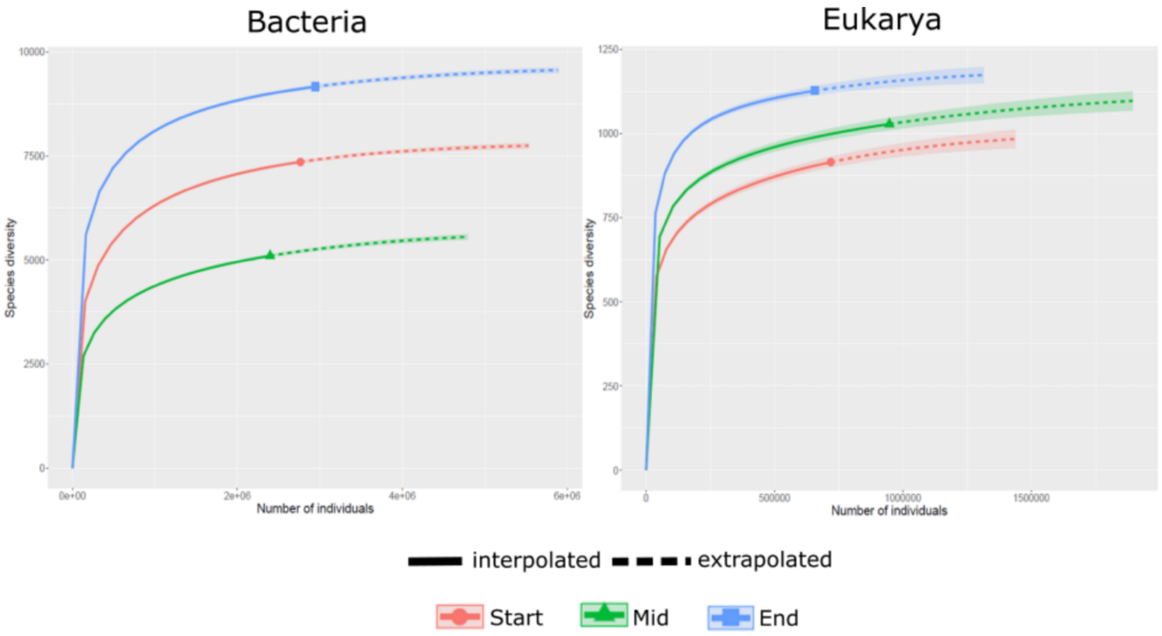

Figure S4. Measurements of robust environmental predictors against soil bacterial communities along the 300 m OW transect (i.e., start = 0-50, middle = 100-150, end = 200-300). The non-uniform distribution of cumulative importance along these gradients indicates that the soil bacterial community responds differently to environmental parameters. Nutrient levels varied across soil community clusters, with mid soils having higher concentrations of Cl^- and Na_2O. For clusters at the start and end, DMF and Fe_2O_3 were in higher concentrations. Dashed lines indicate GF predicted environmental tipping points for taxa at OW, where major shifts in community composition are expected to occur. Significant thresholds were predicted at 100 ppm Cl^-, 3.7% Na_2O, and pH 7.75.

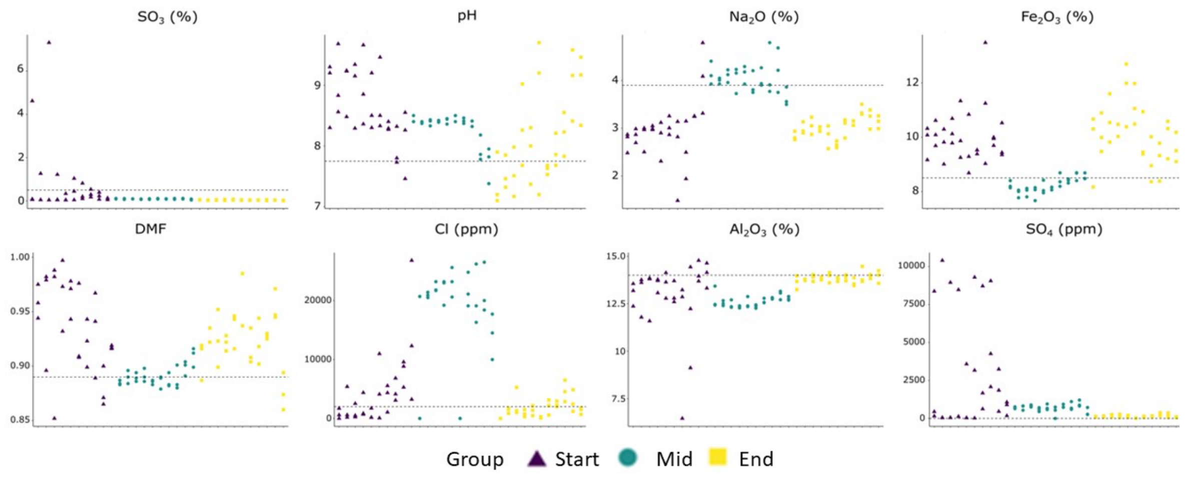

Figure S5. Spearman correlation analysis between taxonomic groups and environmental parameters. Significant correlations ($p < 0.05$) are marked with asterisks and the colour scheme represents the direction of the correlations: blue for positive (Spearman's ρ ranging from 0 to 1) and red for negative correlations (Spearman's ρ ranging from -1 to 0). (A) Correlations at the phylum level, highlighted a significant positive correlation between *Actinobacteria* and DMF, and strong negative correlations between *Acidobacteria* and SO_4^{2-} (Spearman = -0.78), SO_3 and conductivity. (B) At family level, strong positive correlations between *Halomonadaceae* and both conductivity and Na_2O (Spearman = 0.79 for both) were observed. *Nitriliruptoraceae* exhibited strong negative correlations with DMF and positive correlations with conductivity (Spearman = 0.79) and SO_4^{2-} (Spearman = 0.76). Notable negative correlations included *Solirubrobacteraceae* with Na_2O (Spearman = -0.78) and *Gemmatimonadaceae* with conductivity (Spearman = -0.77).

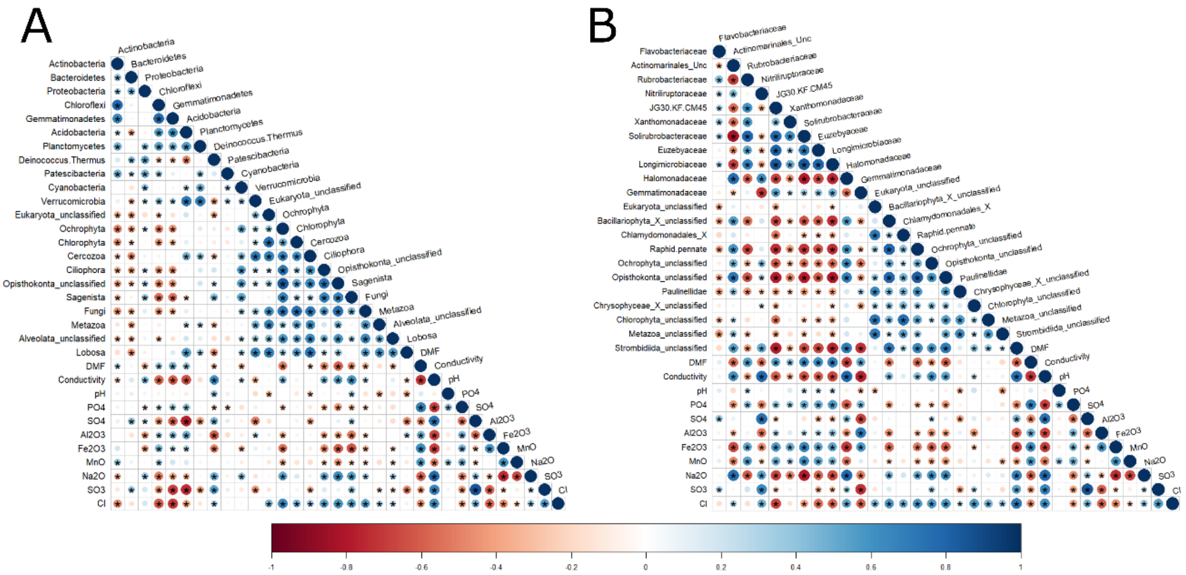

Figure S6. Cumulative plots that visualise bacterial compositional turnover along environmental gradients at Old Wallow, at class level. The y-axis displays cumulative importance on the most responsive taxa, with each coloured line representing a different phylum. Steep slopes or 'splits' indicate high compositional turnover at that environmental threshold. Splits along edaphic gradients were generally non-uniform highlighting variable rates of change in phylum abundances. The top environmental predictors for bacteria at class level were SO_3, SO_4^{2-}, Na_2O, DMF, ASPC, and Cl^- with *Oxyphotobacteria*, and uncultivated taxa (eg Gitt.GS.136, S0134_terrestrial group, PAUC43f_marine_benthic_groupTK10) most responsive.

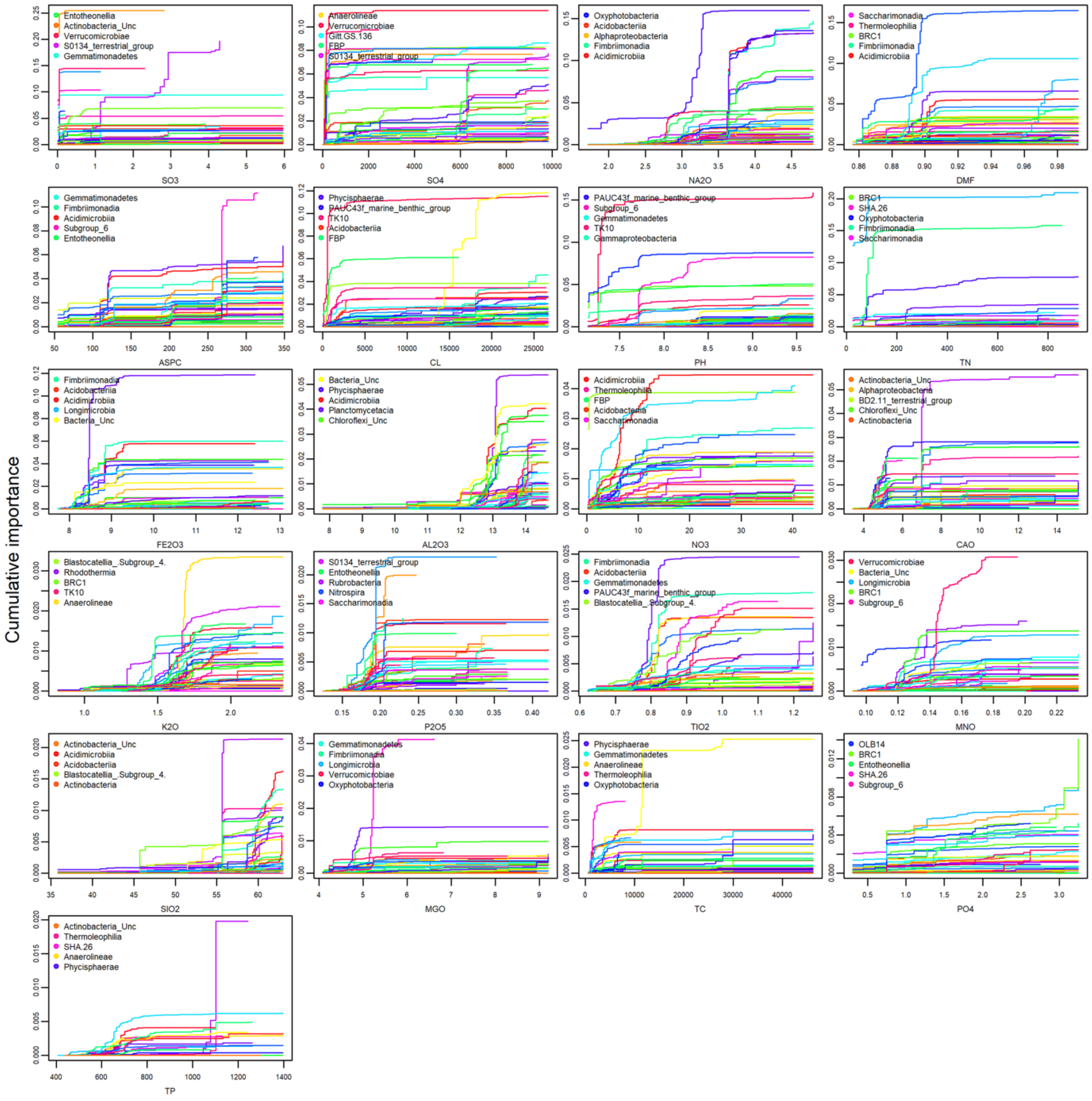

Figure S7. Cumulative plots that visualise eukaryotic compositional turnover along environmental gradients at Old Wallow, at class level. The y-axis displays cumulative importance on the most responsive taxa, with each coloured line representing a different phylum. Steep slopes or 'splits' indicate high compositional turnover at that environmental threshold. Splits along edaphic gradients were generally non-uniform highlighting variable rates of change in phylum abundances. The top environmental predictors for eukaryotes were pH, SO_4^{2-}, SO_3, Na_2O, Cl^- and ASPC. The most responsive classes were largely unclassified taxa – Opisthonkonta__unc, Alveoata_unc, Ochrophyta_unc, Breviatea_X, and MAST12, most of which are associated with marine environments.

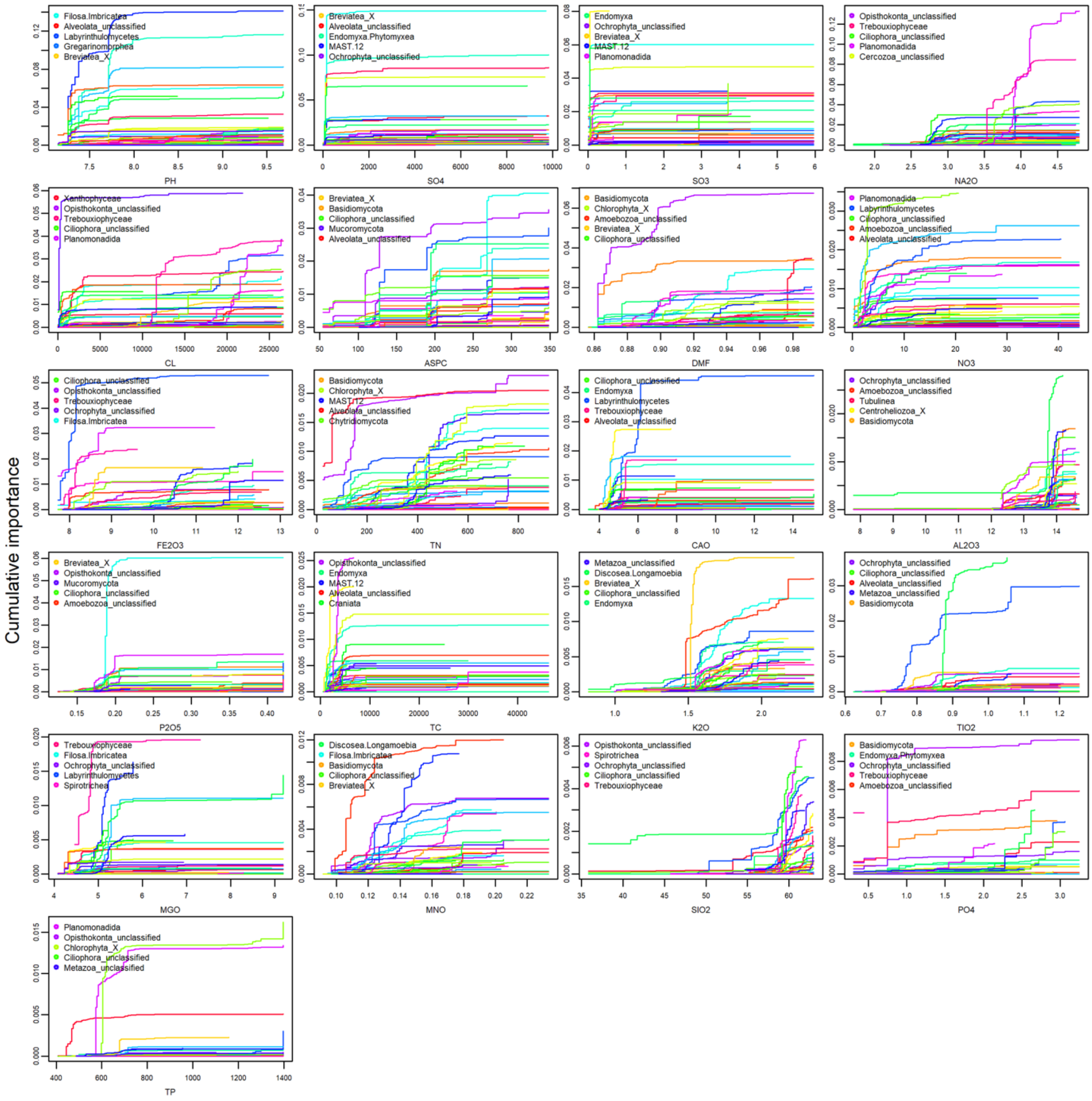

Supplementary Table S1. Environmental Predictors - R2 Weighted Values

Environmental Parameters	Bacteria (R2)	Eukaryotes (R2)
DMF	0.035	0.010
Na_2O	0.030	0.016
Fe_2O_3	0.021	0.007
ASPC	0.019	0.010
Al_2O_3	0.011	0.006
NO_3	0.012	0.007
CaO	0.009	0.006
K_2O	0.007	0.004
SO_3	0.021	0.019
SO_4^{2-}	0.023	0.025
Cl^-	0.024	0.011
pH	0.017	0.026
TiO_2	0.005	0.003
SiO_2	0.004	0.001
PO_4^{3-}	0.002	0.001
P_2O_5	0.004	0.006
MnO	0.003	0.002
TN	0.014	0.007
MgO	0.003	0.003
TC	0.003	0.004
TP	0.001	0.001
